# Monitoring phase transition of aqueous biomass model substrates by high‐pressure and high‐temperature microfluidics

**DOI:** 10.1002/elps.201800431

**Published:** 2019-01-04

**Authors:** Renée M. Ripken, Stefan Schlautmann, Remco G.P. Sanders, Johannes G.E. Gardeniers, Séverine Le Gac

**Affiliations:** ^1^ Applied Microfluidics for BioEngineering Research MESA+ Institute for Nanotechnology and TechMed Centre University of Twente Enschede The Netherlands; ^2^ Mesoscale Chemical Systems MESA+ Institute for Nanotechnology University of Twente Enschede The Netherlands

**Keywords:** Biomass conversion, High‐pressure and high‐temperature, Microfluidics, Multiphase flow, Phase transition

## Abstract

Aqueous‐Phase Reforming (APR) is a promising hydrogen production method, where biomass is catalytically reformed under high pressure and high temperature reaction conditions. To eventually study APR, in this paper, we report a high‐pressure and high‐temperature microfluidic platform that can withstand temperatures up to 200°C and pressures up to 30 bar. As a first step, we studied the phase transition of four typical APR biomass model solutions, consisting of 10 wt% of ethylene glycol, glycerol, xylose or xylitol in MilliQ water. After calibration of the set‐up using pure MilliQ water, a small increase in boiling point was observed for the ethylene glycol, xylitol and xylose solutions compared to pure water. Phase transition occurred through either explosive or nucleate boiling mechanisms, which was monitored in real‐time in our microfluidic device. In case of nucleate boiling, the nucleation site could be controlled by exploiting the pressure drop along the microfluidic channel. Depending on the void fraction, various multiphase flow patterns were observed simultaneously. Altogether, this study will not only help to distinguish between bubbles resulting from a phase transition and/or APR product formation, but is also important from a heat and mass transport perspective.

**Abbreviations**
APR: aqueous‐phase reforming
BPR: back pressure regulator
HPHT: high‐pressure and high‐temperature

## 1 Introduction

Hydrogen has been identified as a promising renewable energy vector to replace fossil fuels in the future 1, 2, 3, in particular when it is produced from biomass 4 using aqueous‐phase reforming (APR). In APR, oxygenated carbohydrates that make up the biomass are reformed into hydrogen in aqueous solution 5. Typically, APR requires elevated temperatures and an external pressure, depending on the exact composition of the reaction mixture, to keep all reagents in the liquid phase 4.

To determine the pressure that is required to maintain the APR reaction mixture in the liquid state, the phase transition of this solution must be known. For a single component the boiling point can easily be calculated as a function of the pressure using the Clapeyron equation. For multicomponent systems, however, the calculation is much more complex, as the components in the solution may interact with each other, which can lead to an increase or decrease in the boiling point. In previous work, we have theoretically determined the boiling point of four APR model reaction mixtures, consisting of aqueous solutions of biomass model substrates (ethylene glycol, glycerol, xylose and xylitol), each as a function of the pressure for mole fractions up to 0.5 using the Redlich‐Soave‐Kwong Boston‐Mathias equation of state 6. We now aim to validate this theoretical model before studying APR experimentally.

To study the phase transition as well as to conduct chemical reactions such as biomass conversion, a microfluidic platform presents numerous advantages: As a result of their high surface‐to‐volume ratio, microfluidic devices provide more efficient heat and mass transfer compared to conventional reactors, excellent flow control, and require only small amounts of sample 7, 8, 9, 10, 11. Several microfluidic set‐ups have been developed to cope with extreme reaction conditions. For example, Tiggelaar et al. 12 proposed a glass‐based microfluidic device with glued‐in capillaries that could withstand pressures up to 690 bar. The Jensen group reported a system to perform catalytic reactions that can withstand both elevated temperatures and pressures, up to 400°C and 300 bar, respectively 13. In the latter device, the pressurized part of the system was thermally insulated from the heated part to prevent the O‐rings used in the connections from melting.

Before studying APR with a similar configuration, we first aim to evaluate the phase transition of model APR reaction mixtures. A number of microfluidic set‐ups have specifically been described for studying phase transitions and for degassing processes 14, 15, 16, 17, 18, 19. Some microfluidic devices contain a constriction in the microchannel, behind which vortices are created. Gas is trapped by these vortices, and bubbles are formed 14, 15. Other systems make use of a local temperature shock 16 or controlled decompression 17 to induce nucleation or of a stop‐flow approach to ensure good control over the multiphase flow 18. In general, a microfluidic format has been proven to be up to 3 times as efficient compared to conventional set‐ups in determining phase transition 14, notably by reducing the time for analysis from hours to minutes 15 as thermodynamic equilibrium is reached quickly.

In this paper, we present a versatile yet simple high‐pressure and high‐temperature (HPHT) microfluidic set‐up, able to withstand temperatures up to 200°C and in which fluids (gas and/or liquid) can be pressurized up to 30 bar. As a first step, we experimentally determined the boiling points of typical APR reaction mixtures, consisting of 10 wt% aqueous biomass model solutions based on ethylene glycol, glycerol, xylose, and xylitol. Knowing the phase transition of these solutions would allow distinguishing between gas product formation and a phase change when conducting APR in these devices. Furthermore, we studied the boiling mechanisms occurring at the microscale and examined how the gas‐liquid flow pattern was evolving depending on the gas fraction in the microchannel. This study will be instrumental in predicting the multiphase flow pattern that is generated from hydrogen gas formation in APR, and how it influences the heat and mass transport in the channel.

## 2 Materials and methods

### 2.1 Microfluidic device

**Figure 1:**
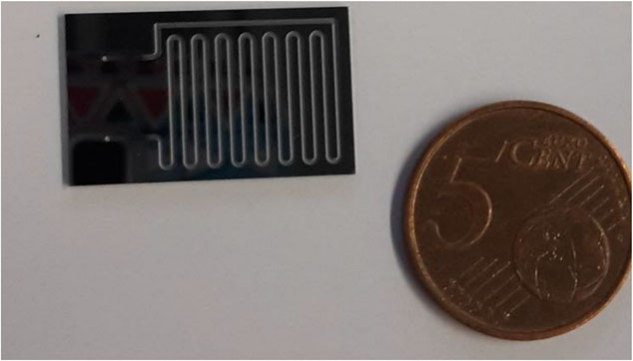
Glass/silicon microfluidic device containing a 0.2 m long meandering channel, which is 500 μm wide and 250 μm deep.

The core of our HPHT set‐up consists of a glass/silicon microfluidic device (Fig. 1). The transparency of the glass top layer allows monitoring the bubble nucleation and the gas/liquid flow regimes during the experiment, whereas silicon ensures good heat transfer to the fluidic channel. The design includes a 500 μm wide, 250 μm deep and 0.2 m long meandering channel with a rectangular cross‐section. The bonding area was optimized, with at least one time the channel width between the meanders, so that the device can withstand pressures up to 30 bar. The device was fabricated using standard microfabrication techniques. Briefly, after a photolithographic step, the channel was dry‐etched (−40°C, 10 mbar He pressure, SF_6_ flow rate 500 sscm, C_4_F_8_ deposition 175 sscm, Adixen, AMS 100) into the silicon substrate (10‐cm diameter, <100>, p/boron‐doped, 525 μm thick, Okmetic). Using the same etching technique the inlet and outlet were machined from the backside of the silicon substrate. Subsequently, a 500 nm SiO_2_ layer was grown on the silicon substrate by wet oxidation at 1150°C for 26 min before it was anodically bonded to the glass substrate (MEMPax, 10 cm diameter, 500 μm thick, Schott AG).

### 2.2 HPHT Set‐up

**Figure 2:**
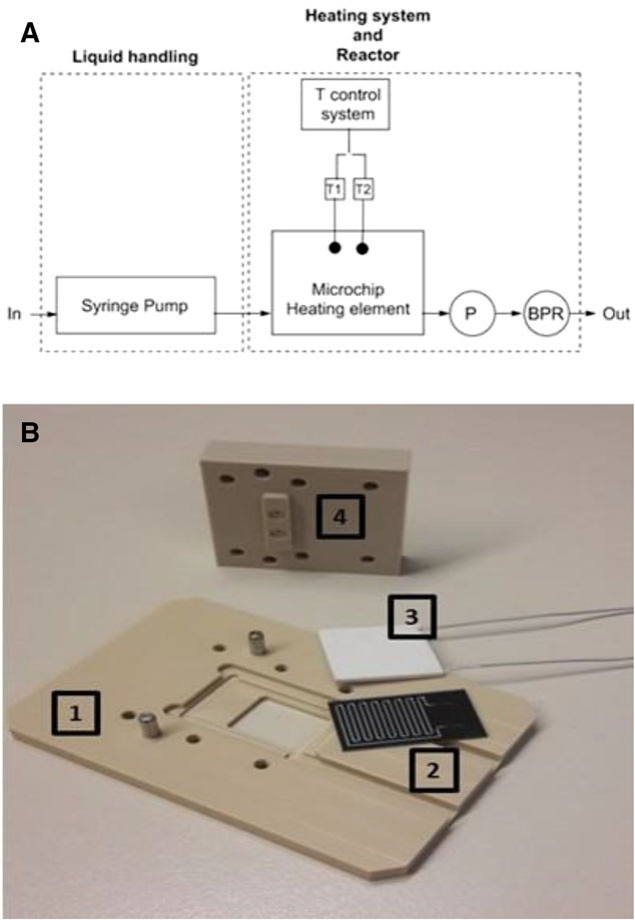
HPHT set‐up. A. Schematic representation of the complete set‐up consisting of a liquid handling part and a chip holder, integrating the microfluidic device and the heater. P = pressure meter; BPR = Back Pressure Regulator. B. PEEK chip holder with 1. Bottom plate with optical window, 2. Microfluidic device, 3. Ceramic heater, 4. Top plate with fluidic connections.

The entire HPHT set‐up consists of two parts: a liquid handling system and a dedicated chip holder for the above described microfluidic device (Fig. 2). The chip holder, which includes both the temperature control and the fluidic connections, was CNC machined from PEEK, a durable and chemically inert material with good thermal insulation properties, which therefore minimizes heat loss to the surroundings. The material properties of PEEK determine the maximum working temperature of the set‐up, which is 250°C in this case. The bottom plate of the chip holder contained an opening, acting as an optical window for real‐time monitoring of the multiphase flow pattern. A ceramic heater (HT24S 24W, Thorlabs) was placed underneath the microdevice at the silicon side and connected to a temperature feedback loop. The temperature was regulated and monitored by two K‐type thermocouples (CHAL‐002, OMEGA) placed between the heater and the silicon side of the device: one for the feedback to the heater and one for the temperature read‐out. The thermocouples were first calibrated and the homogeneity of the temperature field in the microfluidic device was investigated using a thermal camera (FLIR ONE Gen 3 Pro – IOS, resolution 0.1°C).

The pressure was regulated by back pressure regulators (BPR) of 75 (5.2 bar) or 100 psi (6.9 bar) maximum back pressure (IDEX Health & Science) that were connected at the outlet of the chip holder. A pressure meter was included between the outlet of the microfluidic device and the BPR to monitor pressure fluctuations during the experiments.

The fluidic connections between the inlet and outlet of the device and the top plate of the chip holder consisted of silicone O‐rings (ID 1.07 mm; OD 1.27 mm, ERIKS) and PEEK ferrules (F‐126H, Upchurch). PEEK tubing (ID 125 μm; OD 1/32 inch, Inacom) was used to transport the fluids in and out of the system. The liquid was infused by a syringe pump (Chemyx Inc. Fusion 100) and a gastight 250 μL glass syringe (Hamilton).

### 2.3 Phase transition studies and multiphase flow regimes experiments

Four solutions of 10 wt% biomass in MilliQ water were prepared, 10 wt% being the concentration typically used in APR 4, 20. Ethylene glycol, glycerol, D‐xylose and xylitol (Sigma‐Aldrich) were used as received. MilliQ water without any substrate acted as a reference to calibrate the system. The solutions were introduced into the system at a flow rate of 10 μL/min. For each measurement the pressure was fixed by the BPR, while the temperature was increased by 5°C/min starting from room temperature until boiling was observed. The BPR was omitted in the experiments in which the boiling point at atmospheric pressure was determined. The temperature and pressure were monitored during the complete duration of the experiment. A small video camera (DNT, DigiMicro Mobile, maximum 30 frames/s) was used to determine when boiling occurred and to visualize the resulting multiphase flow in the microchannel. Data for each biomass solution were recorded in triplicates.

## 3 Results and discussion

A straightforward, relatively simple system was developed for high‐temperature high‐pressure experiments up to 250°C and at least 30 bar. Our plug‐and‐play set‐up allows quickly changing microfluidic devices, so that the microreactor can be easily adapted to the requirements of the experiment. The microfluidic device in this work can not only be used to determine thermodynamic properties, and to determine the phase transition of mixtures, but also for conducting chemical reactions, such as APR, at real operating conditions, as used in industrially relevant processes. While our set‐up based on PEEK is suitable to conduct experiments at temperatures up to 250°C, the same chip holder design could be realized in, for instance stainless steel, for experiments at even higher temperatures.

### 3.1 Temperature calibration

**Figure 3:**
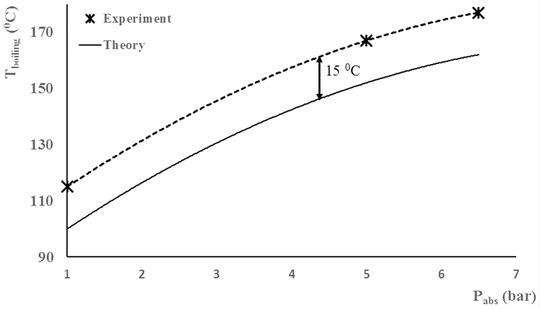
Calibration of the HPHT set‐up with pure MilliQ water. The dashed line corresponds to the experimental results from this work, while the solid line represents theoretical data.

**Figure 4:**
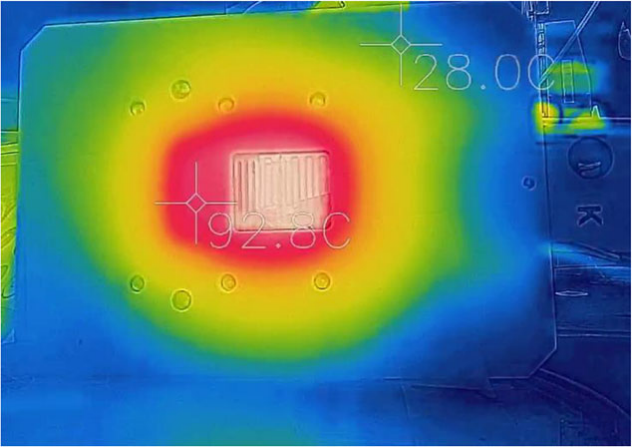
Temperature distribution in the set‐up (bottom plate of the chip holder with microfluidic device) visualized using a thermal camera. Here, temperatures are ranging from 28 (green) to 100°C (white).

According to previous work 21, 22, 23, 24, 25, the temperature measured outside the microfluidic device can significantly differ from the temperature inside the fluidic channels, which is typically caused by a difference in thermal conductivity between the bulk material and the fluid 25. Therefore, the HPHT set‐up was first calibrated with pure MilliQ water. At a flow rate of 10 μL/min the thermal entrance length was found to be negligible (<1 mm), so that the fluid reached almost instantaneously the temperature of the heater. Comparison of the experimental boiling points (Fig. 3, dashed line) with theoretical values (Fig. 3, bold line) revealed a systematic offset of 15°C, which is in the same range as previously reported by Dodge et al. for a similar set‐up comprising an ITO heater 24. All experiments described in this paper have been corrected for this offset. Next, we evaluated the temperature distribution in the device using a thermal camera: a homogeneous temperature field was found (Figure 4), and within a matter of seconds the desired temperature was reached in the entire microfluidic device.

### 3.2 Experimental boiling points and comparison with theoretical data

**Figure 5:**
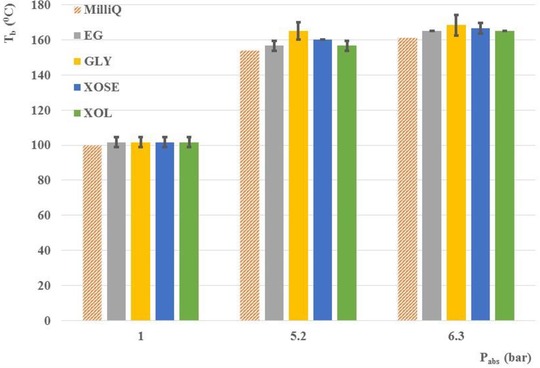
Experimental boiling points of 10 wt% ethylene glycol (EG, grey), glycerol (GLY, yellow), xylose (XOSE, blue) and xylitol (XOL, green) in MilliQ water as a function of absolute pressure. The theoretical boiling point of pure MilliQ water is indicated in the dashed orange bar. The boiling point of each substrate was measured in triplicate.

After calibration of the HPHT set‐up, the liquid‐to‐gas phase transition for the four 10 wt% model substrate/water solutions was studied. The results are presented in Fig. 5, where the dashed orange bar represents the theoretical boiling point of pure MilliQ water. For each substrate solution, the phase transition temperature was measured in triplicate. Addition of 10 wt% of ethylene glycol, xylose and xylitol to water systematically increased the boiling point by 5°C compared to pure MilliQ water, whereas our theoretical model predicted an increase of only 1–2°C 6. Surprisingly, the addition of glycerol did result in an even higher boiling point than expected with less reproducibility than for the other three substrates.

This small yet unexpected difference of 5°C is unlikely to be caused by a pressure drop in the microchannel, which can be calculated by the Darcy‐Weisbach equation:

(1) $$\frac{\Delta P}{L}=\frac{128}{\pi }\frac{\mu }{D^{4}}Q$$

With *L* the length of the channel, *D* its hydraulic diameter, *μ* the dynamic viscosity and *Q* the volumetric flow rate. When using MilliQ at room temperature the pressure drop over our 0.2 m channel is 38.6 Pa, which is negligible. As the viscosity decreases when increasing the temperature, the pressure drop is even lower at higher temperatures.

Another possible explanation could be the differences in the physical properties between the biomass solutions and pure water, since these properties affect the heat transfer to the fluid. The heat transferred is described by:

(2) $$Q=hA\left(T_{wall}-T_{bulk}\right)$$

With *Q* being the total heat transferred and *A* the area through which heat is transported. For a system operating under laminar flow conditions, the heat transfer coefficient *h* at a given location *x* in the channel can be expressed as:

(3) $$h=\frac{Nu_{x}k}{x}=0.332Re_{x}^{1/2}Pr^{1/3}$$

With *Nu_x_* the dimensionless Nusselt number at a given location *x* along the channel, *k* the thermal conductivity of the fluid and *Re* and *Pr* the Reynolds and Prandtl numbers, respectively. As our APR model solutions contain very low substrate concentrations, *Re* is not expected to change significantly compared to pure water. *Pr*, however, depends not only on the viscosity *μ* and the heat capacity *C_p_*, but also on the thermal conductivity *k* according to:

(4) $$\mathrm{Pr}=\frac{\mu C_{p}}{k}$$

Consequently, *Pr* varies more significantly with the solution composition. Therefore, the heat transfer coefficient of the biomass solutions is mainly changing as a result of the change in thermal conductivity of the solution. For example, a 10 wt% solution of ethylene glycol in water has a thermal conductivity of 393 mW/m/K at 1 bar and 99.6°C 26, compared to 677 mW/m/K for pure water under the same conditions [https://www.engineeringtoolbox.com/water-liquid-gas-thermal-conductivity-temperature-pressure-d_2012.html]. As a result, the heat transfer coefficient of the model solutions is lower than that of pure water, which explains the slightly higher offset value we observed for the biomass solutions, which is in the order of the observed 5°C. However, this difference in heat transfer can still not account for the higher boiling point of glycerol, as the thermal conductivity of pure glycerol is comparable to that of pure ethylene glycol (285 mW/m/K for ethylene glycol 26 against 258 mW/m/K for glycerol [https://www.engineeringtoolbox.com/thermal-conductivity-liquids-d_1260.html]). More research is thus required to explain these differences. Taking into account the additional offset as a result of the difference in thermal conductivity, and correcting the experimental boiling points accordingly, it can be concluded that for APR experiments with ethylene glycol, xylose, and xylitol at a concentration of 10 wt% the required pressure to keep the system in the liquid phase can be approximated by that required for pure water.

It should be noted that a 10 wt% substrate solution, depending on the molecular weight of the substrate, corresponds to only a very small mole fraction between 0.01 and 0.03, which is caused by the large difference between the molecular weight of water and the substrate molecule. Based on our previous work 6, such a small mole fraction of biomass would not significantly affect the boiling point compared to pure water, which is in agreement with the experimental results presented here for ethylene glycol, xylose and xylitol.

To further validate the theoretical model a wider range of substrate mole fractions or higher weight percentages should be considered, as a higher biomass content would increase the boiling temperature, according to our theoretical model 6. Specifically, a biomass mole fraction of 0.5 would increase the boiling point by 8 to 24°C, depending on the substrate, which corresponds to solutions with weight percentages up to 90%. Using such high concentrations of biomass would, however, not only change the boiling point, but also other physical properties of the solution, such as the aforementioned thermal conductivity and viscosity, which would significantly affect the transport phenomena in our microfluidic system. At the same time, such high biomass concentrations are less relevant to study, since APR solutions typically contain 10 wt% of the substrate. At higher weight percentages, carbon deposition becomes significant in APR, as well as other side reactions such as methanation, which are detrimental for the hydrogen yield 20.

### 3.3 On‐chip boiling mechanisms: explosive boiling and nucleate boiling

**Figure 6:**
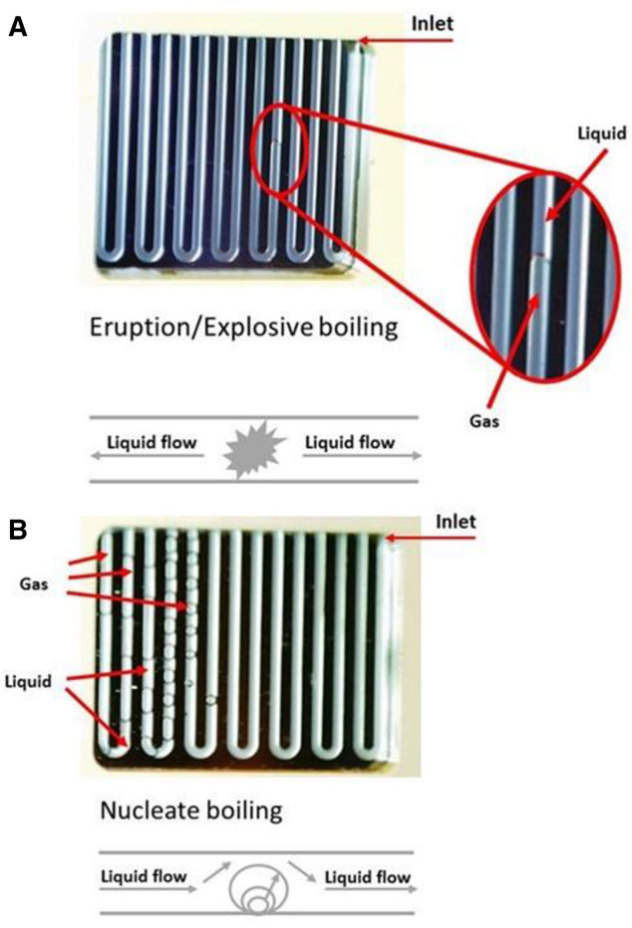
Observed boiling mechanisms. In eruption or explosive boiling (A) the channel is completely filled with gas behind the nucleation site. In nucleate boiling (B) individual gas bubbles are formed.

When studying the phase transition of these biomass model solutions, two boiling mechanisms were observed in the microfluidic device: eruption or explosive boiling (Fig. 6A) and nucleate boiling (Fig. 6B) (See also the movie in Supporting Infomation 1). Explosive boiling was most often observed in our experiments, which is in good agreement with Zhang et al., who reported that this boiling mechanism is predominant in microchannels 27, in particular for systems with a small Péclet number 28.

Typically, in the case of explosive boiling, the liquid was pushed away from the nucleation site both in and against the liquid flow direction. After boiling was achieved, the flow direction was temporarily reversed and the channel was partly cleared from any liquid. We also witnessed a fluctuating gas/liquid front, as already described in the literature (See also the movie in Supporting Information 1) 28.

The resulting gas/liquid multiphase system was highly uncontrollable in terms of flow rate and pressure. Every bubble nucleation caused a local pressure increase of several bar, as a result of the Laplace pressure that has to be overcome before a bubble could be formed. The pressure in the system, which initially increased just before bubble nucleation, subsequently dropped by up to 4 bar and kept fluctuating during boiling. Furthermore, when the gas fraction in the fluidic channel increased, the liquid plug velocity accelerated to fulfill the law of mass conservation. Altogether, the flow rate and pressure continuously fluctuated when saturation was reached.

The second boiling mechanism, nucleate boiling, is typically promoted by the presence of contamination on the microchannel wall or solid particles in the bulk of the liquid. Defects on the microchannel walls also promote this boiling mechanism, which explains the observed nucleate boiling in our set‐up. The method of fabrication of our microchannels, deep reactive ion etching, is a cyclic process that forms small “scallops” at the inner walls of the etched structures, which can act as nucleation sites. In theory, after nucleation, the bubble grows until the buoyancy forces become greater than the interfacial tension forces at the microchannel wall 29. In our experiments, however, the bubble seemed to grow until it reached a size equal to that of the microchannel width, i.e., 500 μm. The bubble subsequently detached when the liquid pressure behind the bubble was high enough to expel it from its nucleation site. Only when the temperature in the microchannel was far above the boiling temperature, the bubble was released at a smaller size. In the case of nucleate boiling, the pressure in the system increased by up to 2 bar before it dropped below the pressure set by the back pressure regulator after the first bubble was formed. In contrast to eruption boiling, the gas/liquid multiphase system was stable and yielded an alternating gas/liquid flow pattern, as discussed in the next section.

### 3.4 Controlling the bubble nucleation site and multiphase flow patterns

**Figure 7:**
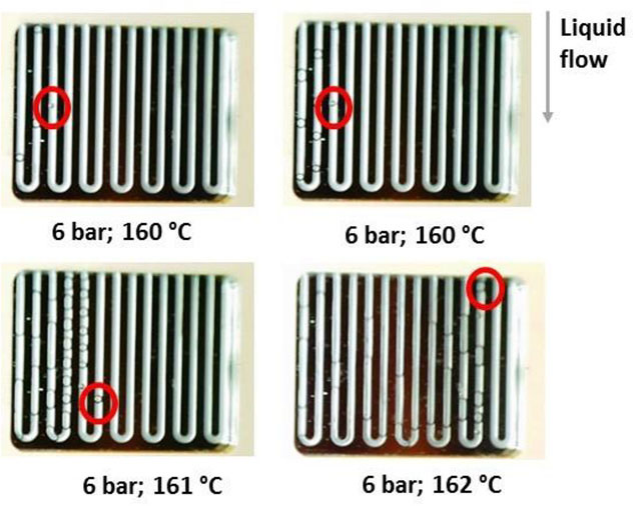
Nucleation site shifting towards the inlet when the temperature is increased from 160°C to 162°C under a constant pressure of 6 bar.

When nucleate boiling occurred, the location in the microchannel where the bubble(s) formed could be controlled by taking advantage of the pressure drop along the channel, resulting from the flowing liquid. Concretely, when the temperature was slowly increased, the nucleation site shifted further upstream towards the inlet of the microchannel, where the pressure was higher. In other words, for a bubble to nucleate closer to the inlet, a temperature increase of only tenths of degrees was required to overcome the higher pressure closer to the inlet (Fig. 7).

The transition from a homogeneous liquid phase to a gas/liquid system, as takes place during boiling, can be translated to the APR process where gaseous products form in a liquid phase. The formation of bubbles and multiphase flow patterns highly influence heat and mass transport and, in turn, the efficiency of the reaction. Therefore, we also studied the evolution of the two‐phase flow and the transition between the different multiphase flow regimes. The resulting gas/liquid flow after saturation was found to be strongly dependent on the gas fraction. In this work, we define the gas or void fraction as the chordal void fraction, which is the ratio between the lengths of the gas plugs divided by the total considered flow length:

(5) $$\alpha_{chordal}=\frac{length_{gas}}{length_{gas\;plugs}+length_{liquid\;plugs}}$$

**Figure 8:**
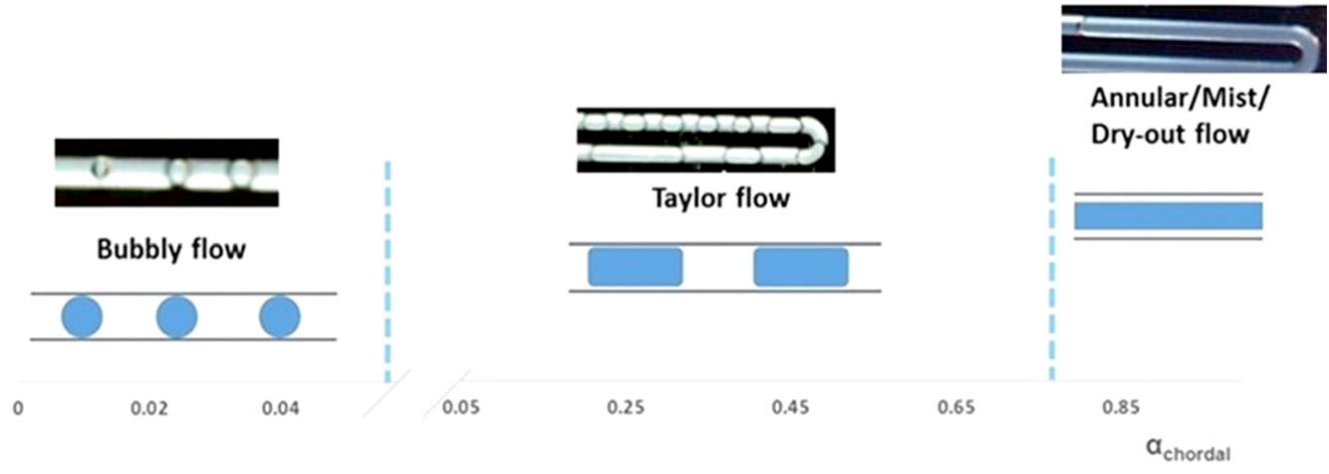
Observed flow regimes as a function of the chordal void fraction. From left to right bubbly flow, Taylor flow and a high void fraction flow regime, which may consist of an annular, a mist and/or a dry‐out flow.

Three different flow regimes were observed depending on the void fraction: (i) bubbly flow, (ii) Taylor flow and (iii) a high void fraction flow regime, which could correspond to an annular, a mist and/or a dry‐out regime (Figure 8). The flow pattern is considered to be bubbly until the size of the gas bubble equals the hydraulic diameter of the microchannel. A Taylor flow is characterized by gas plugs, whose length is greater than the channel width.

The void fraction directly related to the observed flow regimes, as well as the transition from one flow pattern to another one (Figure 8). Multiple flow regimes were observed simultaneously in a single device over the entire void fraction range. The transition from a bubbly flow to a Taylor flow occurred at a void fraction of circa 0.05, whereas the transition from a Taylor regime to the high void fraction flow took place at a void fraction of 0.8, which is in accordance with the literature 17. In the Taylor flow regime, the gas plugs expanded as a result of the pressure decrease along the channel. Furthermore, boiling also occurred further downstream from the nucleation site, creating additional gas, which is taken up by the already existing gas plugs. At the same time, the velocity of the fluid plugs increases downstream to fulfill the conservation of mass. In non‐boiling gas/liquid systems in a bubbly or Taylor flow regime, a thin liquid film of 1–10‐μm thickness 17 exists around the gas phase in the case of hydrophilic microchannel walls. However, in boiling systems it is highly likely that film boiling occurs in this layer, breaking up that thin liquid film 30.

## 4 Concluding remarks

In this work, we reported a versatile high‐pressure and high‐temperature microfluidic set‐up suitable towards studying APR, and which can be operated at temperatures up to 200°C and pressures up to 30 bar. As a first step, we evaluated the phase transition of typical 10 wt% APR biomass model solutions of ethylene glycol, glycerol, xylose and xylitol in MilliQ, after calibrating the system with pure MilliQ water. The addition of three of these substrates in small amounts did not significantly affect the boiling point of pure water. Knowing the phase behavior of the model substrate solutions will allow us in the future to distinguish between a phase transition and the formation of gaseous products during APR. We also characterized the flow regimes resulting from gas generation by either boiling or reaction products by their chordal void fraction. Several flow patterns, from a bubbly flow to combined high void fraction flow regime, were observed simultaneously in a single microfluidic device.

*The authors have declared no conflict of interest*.

## Supporting information

Supporting Information

Supporting Information
